# The Possible Role of the Type I Chaperonins in Human Insulin Self-Association

**DOI:** 10.3390/life12030448

**Published:** 2022-03-18

**Authors:** Federica Pizzo, Maria Rosalia Mangione, Fabio Librizzi, Mauro Manno, Vincenzo Martorana, Rosina Noto, Silvia Vilasi

**Affiliations:** Istituto di Biofisica, Consiglio Nazionale delle Ricerche, Via Ugo La Malfa 153, 90146 Palermo, Italy; pizzofede@gmail.it (F.P.); mariarosalia.mangione@ibf.cnr.it (M.R.M.); fabio.librizzi@cnr.it (F.L.); mauro.manno@cnr.it (M.M.); vincenzo.martorana@cnr.it (V.M.); rosina.noto@cnr.it (R.N.)

**Keywords:** insulin, chaperonins, self-association, amyloid aggregation

## Abstract

Insulin is a hormone that attends to energy metabolism by regulating glucose levels in the bloodstream. It is synthesised within pancreas beta-cells where, before being released into the serum, it is stored in granules as hexamers coordinated by Zn^2+^ and further packaged in microcrystalline structures. The group I chaperonin cpn60, known for its assembly-assisting function, is present, together with its cochaperonin cpn10, at each step of the insulin secretory pathway. However, the exact function of the heat shock protein in insulin biosynthesis and processing is still far from being understood. Here we explore the possibility that the molecular machine cpn60/cpn10 could have a role in insulin hexameric assembly and its further crystallization. Moreover, we also evaluate their potential protective effect in pathological insulin aggregation. The experiments performed with the cpn60 bacterial homologue, GroEL, in complex with its cochaperonin GroES, by using spectroscopic methods, microscopy and hydrodynamic techniques, reveal that the chaperonins in vitro favour insulin hexameric organisation and inhibit its aberrant aggregation. These results provide new details in the field of insulin assembly and its related disorders.

## 1. Introduction

Human insulin is the pivotal hormone for the regulation of glucose levels in the blood. It is produced by the β-cells contained in the pancreatic islets of Langerhans, and its secretion process is a complex metabolic pathway involving several cell compartments and post-translational modifications [1]. Insulin biosynthesis starts within the cytosol, where its mRNA is translated into the precursor preproinsulin (PPI) containing the N-terminal signal peptide (SP), which drives the protein from the cytosol to the endoplasmic reticulum (ER). After being released into ER, by the removal of the SP, PPI converts into proinsulin (PI), a three chains (A, B and C) protein that in the ER lumen undergoes its proper folding. The well-folded PI exits from the ER and translocates to the trans-Golgi network, where it is stored into small compartments called immature granules (IG) [1]. When one of the proinsulin chains, the C-peptide, is removed from proinsulin, the two remaining regions, the A and B chains, connected to each other by two disulphide bonds, form the 51-amino acids insulin protein. The mature granules (MG) resulting from the proinsulin conversion into mature insulin are then secreted by a regulated exocytosis mechanism to release the hormone in response to glucose [1]. Since the first pioneering study on the crystal structure of insulin carried out by the Hodgkin research group [2], a hexamer complex coordinated by zinc ions has emerged as the oligomeric form adopted by the hormone and its precursor proinsulin [3] into the storage vesicles. In particular, two Zn^2+^ ions were identified in the hexamer structure, being coordinated by the three HisB10 imidazole side chains. Electron microscopy on secretory granules from isolated rat Langerhans islets revealed that insulin hexamers are able to further assemble, forming crystal structures [4] with a potential role in protecting insulin from proteolytic enzymes action [1]. Notably, a variant insulin in rodent Octagon degu, which lacks the critical histidine (HisB10) coordinating zinc ions in hexamers, is associated with islet amyloidosis due to insulin fibrillation [5]. Therefore, a potential protective role against insulin pathological aggregation could be attributed to the oligomeric hexamer, whose stability makes it the preferential conformation in drug formulations [6]. The stabilisation of hexamers is crucially important in insulin pharmaceutical preparations, and it is also achieved by regulating the parameters that most influence the hormone oligomeric equilibrium, such as pH, zinc [7] and protein concentration [8,9]. As a matter of fact, while insulin amyloidosis is rarely encountered in mammals, several insulin-derived amyloidoses at the injection site have been associated with diabetes mellitus [10]. Several studies in vitro contributed to understanding the molecular basis of the insulin amyloid self-assembly process and clarifying the role of environmental *factors* in the insulin fibril formation kinetics [11,12,13,14,15,16,17,18,19]. In particular, both insulin aggregation rates and fibril morphologies are strongly influenced by the solution pH; at an acidic pH, the process starts from a homogeneous monomer/dimer population, while at increasing pH values, the equilibrium shifts towards higher molecular weight oligomers, whose dissociation may be rate limiting or partially rate limiting [20,21].

An interesting hypothesis on the insulin hexamers formation in vivo was raised by the experimental evidence of the involvement, in the insulin processing pathway, of the 60 kDa human group I chaperonin, cpn60 [22,23,24]. The presence of the cpn60 was revealed in the secretory pathway of the rat pancreatic insulin-secreting cells, including mature and immature granules, and it showed the capacity of the cpn60 to bind both insulin and proinsulin in vitro [22]. In addition, several studies showed an involvement of cpn60 in diabetes mellitus conditions [25,26]. Soltys and Gupta suggested that, within the granules, cpn60 could possibly have a role in core protein condensation, that is, the formation of hexamers and higher-order oligomers [24]. The hypothesis correlates well with the established role of mammal cpn60 and its bacterial homologue GroEL in the formation of oligomeric protein complexes [27,28]. In fact, by an allosteric mechanism involving the cofactors cpn10 and ATP, 60 kDa proteins belonging to the chaperonins subgroup I of molecular chaperones help polypeptide chains, including themselves, to correctly fold and then assemble in the appropriate oligomeric structures [29,30]. However, apart from these hypotheses, no study has been ever performed to understand the potential role of group I chaperonins in insulin oligomeric assembly within the storage vesicles. Here, we investigated with biophysical methods the ability of the human cpn 60 bacterial homologue GroEL and its cochaperonin GroES to promote the formation of insulin hexamers. Moreover, in light of recent studies demonstrating the chaperonins group I protective action against amyloid aggregation [31,32,33,34], we evaluated the effect of GroEL/GroES on insulin fibrillogenesis at neutral pH. Our results indicate that cpn60 shifts the insulin oligomeric *equilibrium* towards the hexameric form and, as a consequence, inhibits the amyloid fibril formation process. Our findings offer a new perspective in the knowledge of the factors influencing insulin processing and secretory cell pathways with implications in the field of insulin-related disorders and insulin-based therapies.

## 2. Materials and Methods

### 2.1. Materials

Human zinc-free insulin was obtained by Novo Nordisk A/Z Denmark. Mixture from Escherichia coli Chaperonin 60: Chaperonin 10, 1:1 *(*C7563*)* was obtained by Sigma-Aldrich Co. (St. Louis, MO, USA). Zinc chloride (Z0152) and all other chemicals were purchased at analytical grade from Sigma-Aldrich Co. (St. Louis, MO, USA).

### 2.2. Insulin Preparation and Amyloid Aggregation

Insulin stock solutions were prepared by dissolving powdered insulin at ~10 mg/mL in a small amount of 0.1 M acetic acid (pH 2.4), then filtered through 0.22 µm filters. After having determined the protein concentration by using the molar extinction coefficient of 1.0675 cm^−1^ (mg/mL)^−1^ [17], ZnCl_2_ was added (from a 0.16 M stock solution in 0.1 M acetic acid) to the insulin solution. Supposing its full dissociation, zinc chloride was added to the solution at the concentration necessary to obtain 2Zn^2+^ for six insulin monomers. This insulin stock solution was then diluted to reach the concentration required for the experiments into a buffer, from now on referred to as *refolding buffer*, consisting of 100 mM KCl, 5 mM MgCl_2_ and 0.1 mM ATP in 50 mM Tris, pH 7.5 [35].

The powder mixture GroEL/GroES was dissolved in the refolding buffer and, when required, incubated with insulin at a molar ratio GroEL: insulin = 1:10. After incubating insulin and GroEL/GroES mixture, we waited 15 min before starting each of the experiments aimed to study the effect of chaperonins on insulin self-assembly.

For aggregation studies, protein samples at a final concentration of 0.1 mg/mL were incubated at 50 °C under agitation 200 rpm stirring.

### 2.3. Static and Dynamic Light Scattering

The samples were filtered by using 0.22 µm pore size filters into a dust-free quartz cell and kept at 20 °C in the thermostatic cell compartment of a Brookhaven Instruments BI200-SM goniometer. The temperature was controlled within 0.1 °C using a thermostatic recirculating bath. The light scattered intensity and its time autocorrelation function were measured at θ = 90° by using a Brookhaven BI-9000 correlator and a 100 mW solid-state laser (Quantum-Ventus MPC 6000) tuned at wavelength 532 nm. Absolute values of scattered intensity were corrected for the scattering from buffer alone *I*_0_, normalised by the intensity of a toluene standard *I*_tol_ and expressed as Rayleigh ratio via:

(1) $$R_{90}=\left[\frac{I-I_{0}}{I_{tol}}\right]{\left(\frac{n_{s}}{n_{tol}}\right)}^{2}\;R_{90}^{tol}$$

where *n_s_* and *n_tol_* are the refractive indices of the sample and toluene (*n_S_*  =  1.3367 and *n_tol_*  =  1.4996), and the toluene Rayleigh ratio was taken as 28 × 10^−6^ cm^−1^ at 532 nm. Absolute Rayleigh ratio *R*(*q*) is related to the weight averaged molecular mass *M*_w_ of particles by the relation *R*(*q*)  =  *KcM*_w_*P*(*q*), with the instrumental factor *K*  =  4π^2^*ñ*^2^(d*ñ*/d*c*)^2^λ_0_^−4^*N*_A_^−1^, where *c* is the mass concentration, *P*(*q*) is the *z*-averaged form factor, *ñ* is the medium refractive index, λ_0_ is the incident wavelength and *N*_A_ is Avogadro’s number [36]. We calculated the average aggregation number *M*_w_/*M*_0_ = *R*(*q*) (*KcM*_0_)^−1^, with *M*_0_ the monomer molecular mass, by taking (d*ñ*/d*c*) =  (0.18 ± 0.01) cm^3^ g^−1^ and *P*(*q*)  =  1. The form factor is related to the average shape and size of scatterers. However, it is equal to 1 when the size of solutes is much smaller than *q*^−1^ [37].

Due to their Brownian motion, particles moving in solution give rise to fluctuations in the intensity of the scattered light. In a light scattering experiment carried out in dynamic modality, the autocorrelator measures the homodyne intensity–intensity correlation function that, for a Gaussian distribution of the intensity profile of the scattered light, is related to the electric field correlation function:

(2) $$g^{\left(2\right)}\left(q,t\right)={\left[A+Bg^{\left(1\right)}\left(q,t\right)\right]}^{2}$$

where *A* and *B* are the experimental baseline and the optical constant, respectively. For polydisperse particles, *g*^(1)^(*q*,*t*) is given by:

(3) $$g^{\left(1\right)}\left(q,t\right)={\int^{\text{​}}}_{0}^{\infty }G\left(\Gamma \right)exp\left(-\Gamma t\right)d\Gamma$$

Here, *G(Γ)* is the normalised number distribution function for the decay constant *Γ* = *q^*2*^D_T_*, where *q = *(4*πn/λ)sin(θ/*2)** is the scattering vector defining the spatial resolution with *n* and *D_T_* being the solvent refractive index and the translational diffusion coefficient, respectively [37]. The hydrodynamic diameter *D_H_* is calculated from *D_T_* through the Stokes–Einstein relationship:

(4) $$D_{T}=\frac{k_{B}T}{3\pi \eta D_{H}}$$

where *k_B_* is the Boltzmann constant, *T* is the absolute temperature and *η* is the solvent viscosity. *D_H_* was obtained by the intensity autocorrelation functions by means of the method of cumulants [38].

### 2.4. Circular Dichroism Spectroscopy

Circular dichroism (CD) spectroscopic measurements were acquired at 20 °C by using a JASCO J-810 spectrometer (JASCO Corporation, Tokyo, Japan) equipped with a temperature control unit. Quartz cells with 0.2 mm and 10 mm path lengths were used for registering far-UV (190–250 nm) and near-UV (250–300 nm) spectra, respectively. Each spectrum measurement was obtained by averaging over three scans and subtracting the appropriate blank solvent contribution.

### 2.5. Non-Denaturing Gel

Samples were analysed by a non-denaturing gel on a 4–15% gradient (Mini-PROTEAN TGX precast gel, BIO-RAD, USA) and stained with PageBlue protein staining solution (ThermoScientific, Waltham, MA, USA).

Insulin 10 μL aliquots at 0.2 mg/mL and 1 mg/mL in the presence and in the absence of chaperonins were mixed with 1:1 loading buffer (62.5 mM Tris-HCl, 40% (*v*/*w*) glycerol, 0.01% (*v*/*w*) bromophenol blue, pH 6.8). Running buffers: 53 mM Tris, 68 mM glycine, pH 8.9 for cathode (top), 100 mM Tris, pH = 7.8 for Anode (out).

A human neuroserpin with a molecular mass of 46 kDa was used as a reference [39].

### 2.6. Thioflavin T Spectrofluorometric Measurements

Aggregation kinetics was monitored by Thioflavin T *(*ThT) fluorescence by using a JASCO FP-6500 spectrometer. The excitation and emission wavelengths were 450 and 485 nm, respectively, with 3 mm slit widths. ThT concentration was 12 μM. Samples were placed at 50 °C in the thermostated cell compartment (10 mm) and stirred at 200 rpm (magnetic stirred mod. 300, Rank Brothers Ltd., Cambridge, UK).

### 2.7. Atomic Force Microscope (AFM)

Atomic force microscope (AFM) measurements were performed by using a Nanowizard III (JPK Instruments, Berlin, Germany) mounted on an Eclips Ti (Nikon, Tokyo, Japan) inverted optical microscope. Aliquots of protein solutions were deposited onto freshly cleaved mica surfaces (Agar Scientific, Assing S.P.A., Monterotondo, Roma, Italy) and incubated for up to 20 min before rinsing with deionised water and drying under a low-pressure nitrogen flow. Imaging of the protein was carried out in intermittent contact mode in air by using NCHR silicon cantilever (Nanoworld, Neuchatel, Switzerland) with nominal spring constant ranging from 21 to 78 N/m and typical resonance frequency ranging from 250 to 390 kHz.

## 3. Results

We performed two classes of experiments involving the cpn60 bacterial homologue, GroEL, in complex with its cochaperonin GroES. First, we studied the effect of GroEL/GroES on the protein assembly and on the formation of hexameric oligomers, the storage conformations adopted by insulin in pancreatic β cells. Secondly, we triggered the insulin amyloid formation (at 50 °C under stirring) and studied how the fibrillogenesis process was influenced by the presence of GroEL/GroES. In fact, although insulin amyloid aggregation has been well characterised under acidic conditions also at neutral pH, the protein can aggregate, forming amyloid structures [40,41,42]. Both classes of experiments were carried out at pH 7.4 and in a buffer, called *refolding buffer* (100 mM KCl, 5 mM MgCl_2_ and 0.1 mM ATP in 50 mM Tris, pH 7.5) containing all the ingredients necessary for the functioning of the GroEL/GroES molecular machine solution [35].

### 3.1. Zn^2+^ Insulin Self-Association in the Refolding Buffer

#### 3.1.1. Light Scattering

**Figure 1:**
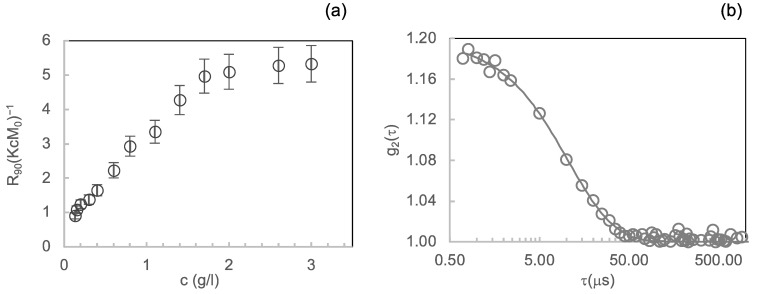
Concentration dependence of insulin oligomerisation state in the refolding buffer monitored by light scattering: (**a**) Scattered light intensities at 90° angle from solutions of insulin protein at different concentrations expressed in terms of the Rayleigh ratio *R_90°_* normalised by (KcM_0_) values; (**b**) second-order autocorrelation functions g_2_ (τ) from dynamic light scattering experiment carried out on insulin at 2 mg/mL. The continuous line represents the fitting curve resulting from data analysis by the method of cumulants.

Insulin assembly into dimers, hexamers and hexameric crystals depends, in vitro and in vivo, on several factors such as pH values, ionic strength, protein and zinc ion concentration [1,8,9,43]. Therefore, as a prior step, we determined the oligomeric composition of insulin at the conditions required for the experiments. In fact, GroEL/GroES-assisted refolding of substrate proteins depends on several factors such as, among others, the concentration of ATP and Mg^2+^ in solution [35]. We determined the insulin oligomeric species equilibrium at varying protein concentrations in the “refolding buffer” (see above). First, we characterised the insulin concentration-dependent association equilibria in the refolding buffer by using light scattering, a technique extensively used to study the associated state of the hormone in solution [8,9,44]. We studied the protein self-association over a range of concentrations, from 0.1 mg/mL to 3 mg/mL, with zinc at a 1:3 Zn^2+^: insulin molar ratio. Figure 1a displays the Rayleigh ratio, normalised by (KcM_0_), at *q*  =  18.7 µm^−1^ (θ scattering = 90°) by insulin at different concentrations as a function of total insulin concentration *c*. Assuming the *z*-averaged form factor *P*(*q*) equal to 1, the *R*_90_(*KcM*_0_)^−1^ results proportional to the ratio *M*_w_/*M_0_*, where *M*_w_ is the average molecular mass of species present in solution and *M_0_* is the monomer molecular mass.

Therefore, the *R*_90_(*KcM*_0_)^−1^ results are proportional to the z-averaged aggregation number. As shown in Figure 1a, *R*_90_(*KcM*_0_)^−1^ values start increasing from 0.15 mg/mL until about 1.7 mg/mL, revealing that under the ionic strength determined by the refolding buffer and in the presence of zinc, a progressive transition from monomers to higher oligomeric species occurs in this range. From 1.7 mg/mL, the *R*_90_(*KcM*_0_)^−1^ values remain almost constantly near ~6 until 3 mg/mL. This suggests the presence of a stable protein oligomeric composition with an average aggregation number of around 6 in the concentration range from 1.7 mg/mL to 3 mg/mL.

To gain further insight into the protein oligomeric equilibrium reached in the concentration region from 1.7 mg/mL to 3 mg/mL, we evaluated by dynamic light scattering (DLS) the autocorrelation function of the light intensity scattered by the protein solution at 2 mg/mL (Figure 1b). We analysed DLS data by using the method of cumulants [38], and we obtained the value of (5.2 ± 0.8) nm for hydrodynamic diameter *D_H_*, the species present in solution. This value is compatible with that reported in the literature for the insulin hexamers diameter [44].

#### 3.1.2. Near-UV CD

**Figure 2:**
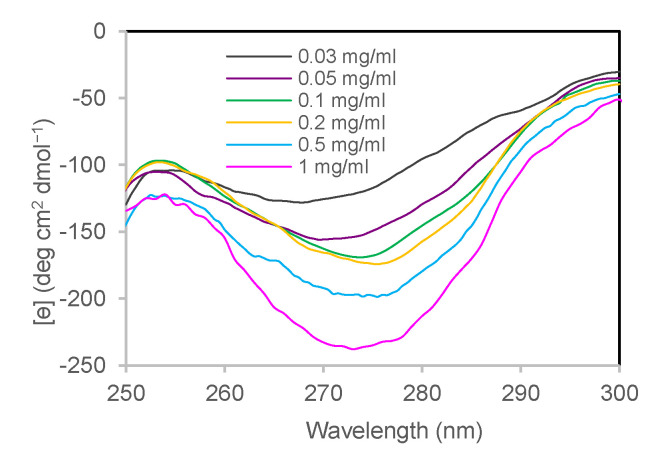
Concentration dependence of insulin oligomerisation state in the refolding buffer monitored by near-UV CD spectroscopy. Near-UV CD spectra of insulin at 0.03 mg/mL (grey), 0.05 mg/mL (violet), 0.1 mg/mL (green), 0.2 mg/mL (yellow), 0.5 mg/mL (light blue), 1 mg/mL (pink).

The state of association of human insulin by varying its concentration was evaluated by circular dichroism in the near-UV region [45,46,47]. A well-marked band in this region’s wavelengths arises from the tyrosine chains present at the interfaces involved in dimer and hexamer formation [47,48]. As reported by Uversky et al., on 20 human insulin mutants with different degrees of association [45], the band intensity is sensitive to the oligomerisation state, being more pronounced for larger-sized oligomers. We recorded near-UV CD spectra for insulin at different concentrations (from 0.03 to 1 mg/mL) obtained by opportune dilution in the refolding buffer at pH 7.4 and in the presence of Zn^2+^ in the molar ratio Zn^2+^: insulin = 1:3. By increasing the protein concentration, the mean residue molar ellipticity presents a deeper band with a minimum at 275 nm, indicating a greater association state (Figure 2).

### 3.2. GroEL Effect on Zn^2+^ Insulin Self-Association

#### 3.2.1. Near-UV CD

**Figure 3:**
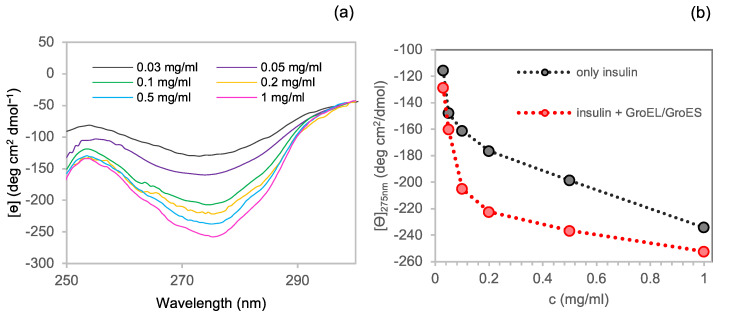
Concentration dependence of insulin oligomerisation state in the presence of GroEL/GroES monitored by near-UV CD spectroscopy: (**a**) Near-UV CD spectra of insulin in the presence of GroEL/GroES and subtracted of the chaperonins corresponding spectra at 0.03 mg/mL (grey), 0.05 mg/mL (violet), 0.1 mg/mL (green), 0.2 mg/mL (yellow), 0.5 mg/mL (light blue) and 1 mg/mL (pink); (**b**) insulin mean residue ellipticity at 275 nm in the presence (red) and in the absence (black) of GroEL/GroES.

The study of the effect of chaperonins GroEL/GroES (1:1) on insulin self-association was performed by considering a 1:10 GroEL to insulin molar ratio [44]. Given that GroEL is a tetradecamer of a 58 kDa monomer, with a multimer molecular mass of~800 kD [49,50,51], light scattering was not fit to study the mix of the two proteins. The GroEL CD signal in the near-UV region result was relatively flat in comparison to the insulin one at the selected chaperonins:insulin molar ratio, as shown in Figure S2 (Supplementary Materials) at one of the tested concentrations. Therefore, the insulin spectrum can be easily obtained by subtracting the CD spectrum of the refolding buffer containing GroEL and GroES from that of insulin and chaperonins mixed together. For this reason, this technique turned out to be the appropriate method to observe the effect of GroEL/GroES on the hormone self-association. Figure 3a shows the CD near-UV spectra of the insulin in the presence of chaperonins and corrected by subtracting the correspondent GroEL/GroES spectra.

In order to highlight the effect of the chaperonins on the CD band amplitude in the near-UV region, we plotted the mean residue ellipticity vs insulin concentration both in the presence and in the absence of GroEL/GroES (Figure 3b). In the presence of chaperonins, insulin produced a deeper CD signal band at each of the tested concentrations, thus suggesting the formation of higher molecular weight species in solution. Therefore, for instance, based on Uversky data [45], while the residue molar ellipticity of insulin at 0.2 mg/mL can be associated with a dimers or trimers conformation, the value at the same concentration in the presence of GroEL/GroES would correspond to an association state near 6, indicating insulin hexamers.

#### 3.2.2. Native Electrophoresis

**Figure 4:**
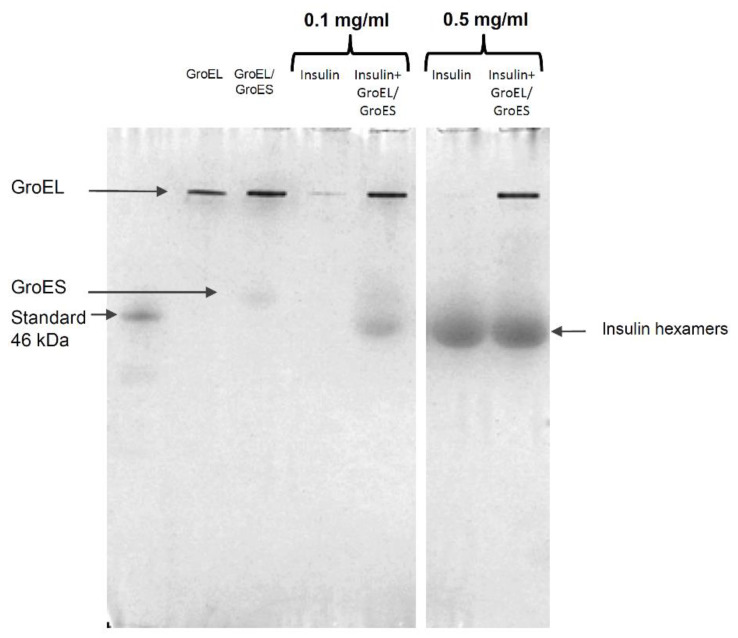
Non-denaturant PAGE of insulin at 0.1 and 0.5 mg/mL with and without GroEL/GroES in a molar ratio GroEL:insulin = 1:10. In addition, GroEL at 0.1 mg/mL (which corresponds to the concentration of GroEL incubated with 0.1 mg/mL insulin at a molar ratio chaperonin/insulin 1:10) and GroEL/GroES mix (GroEL:GroES = 1:1 with GroEL at 0.1 mg/mL) were loaded. The original non-denaturant page is reported in the Supplementary Materials (Figure S1).

A further confirmation of the GroEL/GroES capability to promote insulin hexamers formation was obtained by native page experiments. As shown in the gel image (Figure 4), at a concentration of 0.1 mg/mL, insulin gives rise to a band corresponding to the hexameric structure only when the hormone is incubated with the chaperonins. On the contrary, no band corresponding to hexamers is originated from the insulin sample at 0.1 mg/mL. This finding confirms that, at this concentration, as suggested by near-UV CD, insulin is organised as low molecular weight species that migrate with the solvent front so as not to be retained in the gel meshes. The gel shows that at 0.5 mg/mL, insulin hexamers are present also in the absence of chaperonins, again pointing out the role of concentration in protein oligomeric equilibrium.

### 3.3. Influence of Chaperonins on Zn^2+^ Insulin Amyloid Formation at pH 7.4

#### 3.3.1. ThT Fluorescence Assay

**Figure 5:**
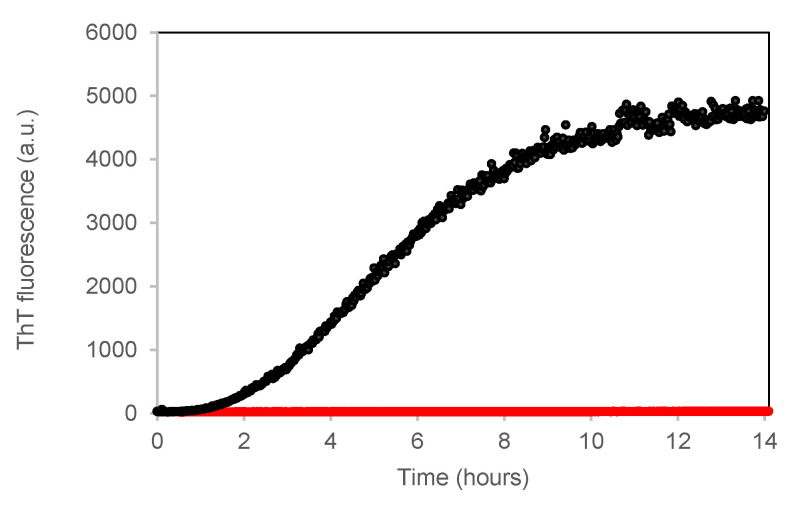
ThT assay to study GroEL/GroES effect on insulin fibrillogenesis at pH 7.4. Time dependence of ThT fluorescence at 482 nm (ex 450 nm) upon incubating the dye (12 μM) with insulin at 0.1 mg/mL, 50 °C and under 200 rpm stirring in the absence (black) and the presence of GroEL/GroES (red) at a molar ratio (GroEL/GroES):insulin = (1:1):10.

Insulin amyloid formation depends on several factors such as pH, concentration, ionic strength, temperature, agitation, as well as different ions and additives [11,12,14,17]. Here, in order to study the GroEL/GroES possible effect on insulin amyloid aggregation, we decided to thermally trigger the process by incubating the sample at 50 °C. Although the fibrils formed at neutral pH appear larger and more frayed when compared with the elongated structures formed at acidic pH and even more thicker in the presence of zinc [41], they are able to bind the thioflavin T (ThT) dye and present the typical cross-β spine structure that characterises the on-pathway amyloid species [14,40,41,52]. We used ThT to study the insulin aggregation kinetics at pH 7.4 at a temperature of 50 °C and under 200 rpm agitation in the absence and in the presence of GroEL/GroES with a fixed molar ratio (GroEL/GroES):insulin = (1:1):10. The fluorescence of ThT incubated with insulin at the beginning of the process follows, in time, a sigmoidal profile, which can be ascribed to the typical nucleation-polymerisation process characterising amyloid formation (Figure 5). After a lag-phase of ~1.5 h long, ThT fluorescence increases for 12 h in response to fibril growth. On the contrary, no significant change in ThT fluorescence could be observed when we incubated the insulin sample with GroEL for up to 14 h, suggesting that amyloid fibrillation is inhibited by the chaperonins’ presence.

#### 3.3.2. Far-UV CD

**Figure 6:**
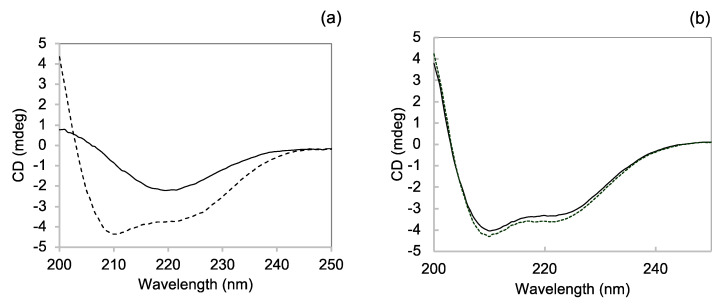
Far-UV CD to assess the influence of GroEL/GroES on the insulin conformational transition accompanying amyloid formation. CD spectra of 0.1 mg/mL insulin at the beginning (dotted line) and after 14 h (continuous line) of incubation at 50 °C, under 200 rpm stirring in the absence (**a**) and in the presence (**b**) of chaperonins.

Direct information on the structure of insulin aggregates formed in the presence of chaperonins was obtained with CD spectroscopy in the far-UV region, which is a very sensitive technique widely used to monitor the α to β transition underlying insulin amyloid formation [53,54]. Figure 6a shows the dichroic spectrum of insulin at the beginning of the amyloid aggregation process. The spectrum presents the two α helix typical minima at 208 and 222 nm. After 14 h incubation, the spectrum shape is dramatically changed, exhibiting a minimum around 220 nm, associated with β-sheet structure elements [53] (Figure 6a). Interestingly, the insulin spectrum in the presence of GroEL after 16 h incubation is almost fully overlapping with the spectrum before incubation (Figure 6b), thus showing that GroEL inhibits the structural conversion accompanying insulin amyloid formation.

#### 3.3.3. Atomic Force Microscopy

**Figure 7:**
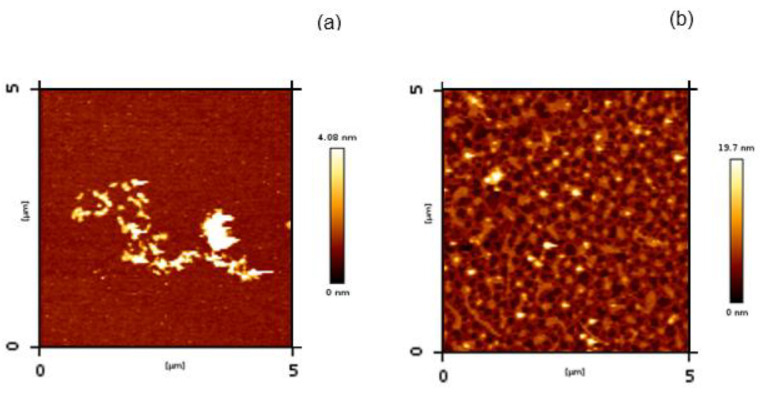
Insulin amyloid aggregation in the absence (**a**) and in the presence (**b**) of GroEL/GroES monitored by AFM. The scale represents the height of pixels in the image. The protein samples were taken 14 h from the beginning of the aggregation process.

We next examined the morphologies of the insulin aggregates obtained in the presence and in the absence (control) of the chaperonins by AFM (Figure 7). As expected for insulin aggregated at neutral pH [21,40], fibrillary material tends to form short–stumpy fibril clusters (Figure 7a), with a different morphology than elongated fibrils typically observed for insulin amyloid species formed at pH 2.2 [14]. In contrast, a granular species dense network is present in the image from the protein sample incubated with the chaperonins.

## 4. Discussion

In this study, we describe how the presence of the chaperonin GroEL and its cochaperonin GroES affect in vitro the oligomeric equilibria of the human insulin in solution. The experiments attempt to explore the possibility that the human cpn60, GroEL mammal homologue, plays a role in insulin hexamers formation, the storage conformation found inside the pancreatic β cells storage vesicles.

The hypothesis, based on the evidence of the cpn60 presence in each of the steps characterising the insulin synthesis and secretory pathways [22,23,24], correlates well with the cpn60 known function to assist folding and assembly of non-native proteins. In fact, both cpn60 and GroEL are large cylindrical oligomers that sequester in their large cavity non-native proteins and release them once they reach their native conformation necessary for their subsequent correct oligomerisation [27,28,30,55,56]. Moreover, while cpn60 was initially thought to primarily function within mammalian cells, a variety of experiments has demonstrated that small amounts of these chaperones may also function elsewhere in the cell [24,49,57].

Some evidence resulting from our experiments suggest that the GroEL/GroES molecular machine could have a role in promoting insulin hexamers formation. The first evidence results from the analysis of insulin incubated at different concentrations in the presence and in the absence of the chaperonins by using near-UV CD. In fact, the near-UV CD signal of insulin has been predicted [46] and experimentally verified to be sensitive to the degree of protein association [45,46]. In particular, the band observed for insulin at 275 nm essentially arises from tyrosyl side chains, three of which are positioned in the external surfaces of the protein directly involved in contacts between monomers to form dimers and dimers to form hexamers. Therefore, at increasing oligomeric size of insulin, new coupling interactions between the tyrosine side chains with the neighbouring molecules and, concomitantly, an enhanced CD ellipticity value at 275 nm can be observed. In our experiments, we manipulated the insulin oligomeric equilibrium by varying the protein concentration in solution. When we registered CD spectra in the near-UV region for insulin at different protein concentrations (Figure 3), we found a value for 275 nm molar ellipticity (~−116 deg cm^2^ dmol^−1^) at 0.1 mg/mL compatible with the presence of monomers/dimers in solution [45] and significantly negatively enhanced at 1 mg/mL with a value (~−234 deg cm^2^ dmol^−1^) that can be associated with hexameric structures [45]. At each of the analysed concentrations, the CD negative band amplitude at 275 nm resulted in an increased presence of GroEL/GroES, revealing a higher state of association for the insulin samples. Non-denaturing gel further confirms that chaperonins are able to shift insulin species equilibrium towards higher molecular weight oligomers. In fact, hexamers were present only when the insulin sample at 0.1 mg/mL was incubated with the GroEL/GroES, whereas no band corresponding to hexameric conformations was observable in the absence of chaperonins (Figure 4).

The different oligomeric compositions between the two insulin samples at 0.1 mg/mL in the absence and in the presence of GroEL/GroES caused, as a direct consequence, different behaviour exhibited by the samples when they were incubated under 50 °C and agitation. In the absence of chaperonins, the only insulin formed, in time, structures that were able to bind ThT, increasing its quantum yield and with a CD spectrum characterised by a beta-sheet single minimum (Figure 5 and Figure 6a). In contrast, no increase in ThT fluorescence or change in CD spectrum was observed in time for the sample incubated with GroEL/GroES (Figure 5 and Figure 6b). We conclude that the amyloid fibril formation resulted completely inhibited in the presence of chaperonins.

Several observations showed how the presence of higher-order oligomeric states in the initial insulin sample is at the basis of a self-inhibition process that determines an unexpected dependence of amyloid aggregation reaction half-times (time at which ThT fluorescence reaches the half-maximum value) on the insulin concentration monomers [58]. Hence, according to the classical nucleation-polymerisation model, whereas at an acidic pH, the insulin sample, which is essentially populated by monomers/dimers, aggregates with a rate that increases with the concentration, at a neutral pH in which higher-order oligomeric states are predominant, the amyloid formation has an opposite behaviour at varying concentrations [21]. Probably the higher-order oligomeric states are amyloid off-pathway conformations that do not participate in fibril elongation (thus reducing the low molecular weight species prone to aggregate) or interfere with it. We can hypothesise that the presence of GroEL/GroES, favouring the formation of higher-order insulin species such as hexamers, induces a similar self-inhibition process in the insulin sample and the amyloid formation results prevented. This confirms again the potentiality of chaperonin type I to protect against protein pathological aggregation processes. In fact, both GroEL and human Hsp60 have been shown to inhibit the amyloid aggregation pathway of the Aβ peptide involved in Alzheimer’s disease [31,32,34,59]. In those cases, the aggregation inhibition effect was exerted by the cpn60 molecules in the absence of their cofactors cpn10, and specific interactions of chaperonins with toxic amyloid Aβ monomers/low molecular weight seeds were invoked. Different from this *holding* action, here, the interference with insulin protein amyloid aggregation is the direct consequence of the *folding* action classically attributed to GroEL. In fact, molecular chaperones have been divided into functional subclasses based on their mechanism of action [60]. Chaperonins, together with other molecular machines such as DnaK, are classified as *folding* chaperones able, in an ATP-driven manner, to induce in their substrates the proper conformational changes to reach the native structure. According to this function, GroEL structurally modifies the insulin to the correct subsequent oligomeric assembly, leading to the formation of stable hexamers and preventing its amyloid aggregation.

A great deal of studies on insulin hexamers describe oligomeric conformation as an allosteric structure capable of undergoing transitions between three conformational states, T_6_, T_3_R_3_ and R_6_, with differences in the secondary structure of the B1–B6 region in the insulin B-chain [61]. While the insulin monomer in solution resembles the T-state, T_3_R_3_ has been hypothesised as the more plausible in vivo conformation [62]. Hence, structural rearrangements involving the insulin chain B terminal region are supposed to occur in cells. However, the molecular mechanisms at the basis of this conformational switch are still unclear. Since monomers/dimers in the R-state have never been observed [63], if we hypothesised that this conformational transition precedes the hexamers formation, it would require an entropic contribution that could be provided by a chaperone molecular machine such as GroEL/GroES. However, this remains, at the moment, only a hypothesis. Further studies, for example, by the combined use of chromatography and X-ray crystallography, are needed in the future to assess the possible contribution of type I chaperonins to the transition from T-state to R-state in the insulin monomer.

## 5. Conclusions

In conclusion, our study demonstrates the capability in vitro of the group I chaperonin GroEL together with its cochaperonin GroES to favour the formation of hormone insulin hexameric structures. In addition, we show how the shift in the insulin oligomeric equilibrium towards higher association states prevents its amyloid aggregation.

Hexamers are the storing conformations adopted by the hormone in pancreatic β cells, and they are the forms preferentially used in pharmaceutical formulations to avoid insulin-derived amyloidosis phenomena in diabetes mellitus patients. Thus, cpn60/cpn10, which are present along each of the secretory pathway steps of insulin, could have a role to assist the fold and assembly of the hormone inside the cells, thus preventing aberrant aggregation. We believe that our results provide new information on the molecular mechanisms at the basis of insulin processing in pancreatic cells and could also offer insights into the diabetes mellitus drugs therapeutic field.

## Data Availability

The data presented in this study are available on request from the corresponding author.

## Supplementary Materials

Page: 16The following supporting information can be downloaded at: https://www.mdpi.com/article/10.3390/life12030448/s1, Figure S1: Original non denaturant PAGE used for composing the figure 4 of the manuscript.; Figure S2: Near-UV CD spectra of insulin at 0.5 mg/ml (86 μM) in the presence of the chaperonins GroEL/GroES in a molar ratio insulin:(GroEL:GroES)=10:(1:1) and uncorrected for the corresponding chaperonins signal (dashed black line). In the same graph, the spectrum relative to GroEL/GroES (GroEL 8.6 μM) is reported (grey), together with the spectrum obtained for insulin after the subtraction of the chaperonins signal from the uncorrected spectrum (continuous black).
