# Supplementary material for: The Possible Role of the Type I Chaperonins in Human Insulin Self-Association
Source: Life (Basel). 2022 Mar 18;12(3):448. doi: 10.3390/life12030448 (PMC8949404; doi:10.3390/life12030448)
Supplement: Supplementary file 1 [file life-12-00448-s001.zip › life-1621676-supplementary.pdf]

life

MDPI

The Possible Role of the Type I Chaperonins in Human Insulin Self-association

Federica Pizzo, Maria Rosalia Mangione, Fabio Librizzi, Mauro Manno, Vincenzo Martorana ,Rosina Noto and Silvia Vilasi

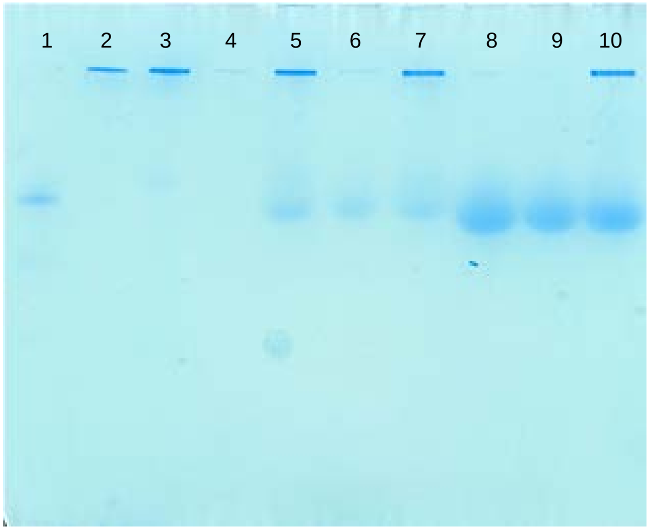

Figure S1: Original non denaturant PAGE used for composing the figure 4 of the manuscript. In particular the columns 1, 2, 3, 4, 5, 9, 10 used for the figure are **1**: a human neuroserpin with molecular mass of 46kDa was used as a reference; **2**: GroEL at 1.7 µM; **3:** GroEL/GroES mixture in a molar ratio 1:1 at 1.7 µM; **4**: insulin at 0.1 mg/ml (17 µM); **5**: insulin at 0.1 mg/ml (17 µM) with GroEL/GroES in a molar ratio GroEL:insulin=1:10. **9**: insulin at 0.5 mg/ml (86 µM); **10**: insulin at 0.5 mg/ml (86 µM) with GroEL/GROES in a molar ratio GroEL:insulin=1:10.

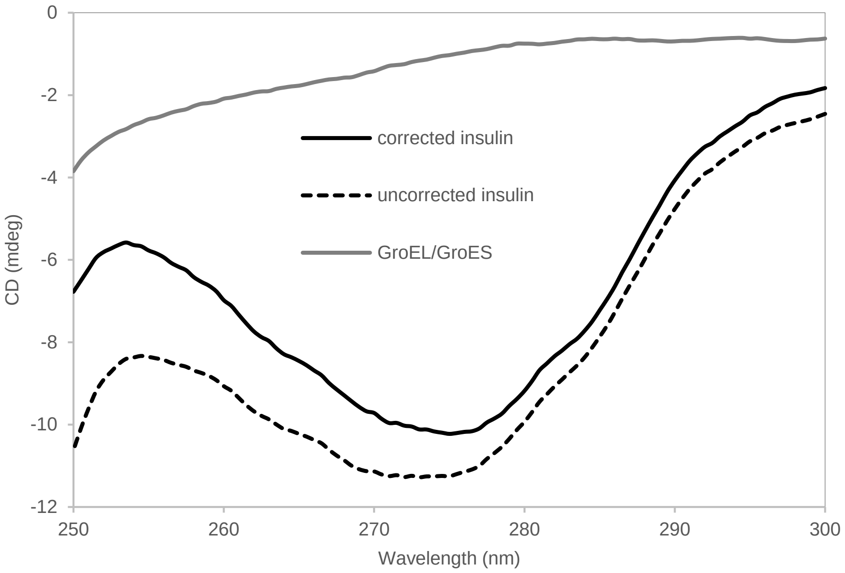

Figure S2: Near-UV CD spectra of insulin at 0.5 mg/ml (86 μM) in the presence of the chaperonins GroEL/GroES in a molar ratio insulin:(GroEL:GroES)=10:(1:1) and uncorrected for the corresponding chaperonins signal (dashed black line). In the same graph, the spectrum relative to GroEL/GroES (GroEL 8.6 μM) is reported (grey), together with the spectrum obtained for insulin after the subtraction of the chaperonins signal from the uncorrected spectrum (continuous black).
